# Supplementary material for: ITAS: Integrated Transcript Annotation for Small RNA
Source: Noncoding RNA. 2022 May 2;8(3):30. doi: 10.3390/ncrna8030030 (PMC9150019; doi:10.3390/ncrna8030030)
Supplement: Supplementary file 1 [file ncrna-08-00030-s001.zip › ITAS_Supplemental_Figures&Tables_legends.pdf]

Supplemental Figure S1. Top differentially expressed small RNA (sRNA) using Donkin et.al., 2016 data, processed with SPORTS vs integrated transcript annotation for small RNA (ITAS) based genome-alignment (bowtie + Rsubread) pipelines.

Supplemental Figure S2. Top differentially expressed sRNA using Ingerslev et.al., 2018 data, processed with SPORTS vs ITAS based genome-alignment (bowtie + Rsubread) pipelines.

Supplemental Figure S3. Top differentially expressed sRNA using Hua et.al., 2019 data, processed with SPORTS vs ITAS based genome-alignment (bowtie + Rsubread) pipelines.

Supplemental Figure S4. Top differentially expressed tRNA-derived small RNA (tsRNA) using Donkin et.al., 2016 data, processed by SPORTS vs ITAS based genome-alignment (bowtie + Rsubread + kallisto) pipelines.

Supplemental Figure S5. Top differentially expressed tsRNA using Ingerslev et.al., 2018 data, processed by SPORTS vs ITAS based genome-alignment (bowtie + Rsubread + kallisto) pipelines.

Supplemental Figure S6. Top differentially expressed tsRNA using Hua et.al., 2019 data, processed by ITAS based genome-alignment (bowtie + Rsubread + kallisto) pipeline. Using SPORTS pipeline, no differential expressed tsRNA were identified.

Supplemental Table S1. Differentially expressed sRNA using Donkin et.al., 2016 data, processed with SPORTS.

Supplemental Table S2. Differentially expressed tsRNA using Donkin et.al., 2016 data, processed with SPORTS.

Supplemental Table S3. Differentially expressed sRNA using Donkin et.al., 2016 data, processed with integrated transcript annotation for small RNA (ITAS) based genome-alignment (bowtie + Rsubread) pipeline.

Supplemental Table S4. Differentially expressed tsRNA using Donkin et.al., 2016 data, processed with ITAS based genome-alignment (bowtie + Rsubread + kallisto) pipeline.

Supplemental Table S5. Differentially expressed sRNA using Hua et.al., 2019 data, processed with SPORTS.

Supplemental Table S6. Differentially expressed sRNA using Hua et.al., 2019 data, processed with ITAS based genome-alignment (bowtie + Rsubread) pipeline.

Supplemental Table S7. Differentially expressed tsRNA using Hua et.al., 2019 data, processed with ITAS based genome-alignment (bowtie + Rsubread + kallisto) pipeline.

Supplemental Table S8. Differentially expressed sRNA using Ingerslev et.al., 2018 data, processed with SPORTS.

Supplemental Table S9. Differentially expressed tsRNA using Ingerslev et.al., 2018 data, processed with SPORTS.

Supplemental Table S10. Differentially expressed sRNA using Ingerslev et.al., 2018 data, processed with ITAS based genome-alignment (bowtie + Rsubread) pipeline.

Supplemental Table S11. Differentially expressed tsRNA using Ingerslev et.al., 2018 data, processed with ITAS based genome-alignment (bowtie + Rsubread + kallisto) pipeline.

Supplemental Table S12. Statistics on correction events in Mus musculus transcript entries: cases when both locus and sequence were present (Loci & Seq, no correction), only locus or only sequence (Loci only, Seq only, sequence or locus retrieved from genome); cases that required extending entry's locus, sequence or both (Ext Loci & Seq, Loci & Ext Seq, Ext Loci & Ext Seq).

Supplemental Table S13. Statistics on correction events in Rattus norvegicus transcript entries: cases when both locus and sequence were present (Loci & Seq, no correction), only locus or only sequence (Loci only, Seq only, sequence or locus retrieved from genome); cases that required extending entry's locus, sequence or both (Ext Loci & Seq, Loci & Ext Seq, Ext Loci & Ext Seq).

Supplemental Table S14. Statistics on correction events in Drosophila melanogaster transcript entries: cases when both locus and sequence were present (Loci & Seq, no correction), only locus or only sequence (Loci only, Seq only, sequence or locus retrieved from genome); cases that required extending entry's locus, sequence or both (Ext Loci & Seq, Loci & Ext Seq, Ext Loci & Ext Seq).

Supplemental Table S15. Statistics on correction events in Caenorhabditis elegans transcript entries: cases when both locus and sequence were present (Loci & Seq, no correction), only locus or only sequence (Loci only, Seq only, sequence or locus retrieved from genome); cases that required extending entry's locus, sequence or both (Ext Loci & Seq, Loci & Ext Seq, Ext Loci & Ext Seq).
