# Supplementary material for: ITAS: Integrated Transcript Annotation for Small RNA
Source: Noncoding RNA. 2022 May 2;8(3):30. doi: 10.3390/ncrna8030030 (PMC9150019; doi:10.3390/ncrna8030030)
Supplement: Supplementary file 1 [file ncrna-08-00030-s001.zip › ITAS_Supplemental_Figures_21022022.pptx]

## Slide 1
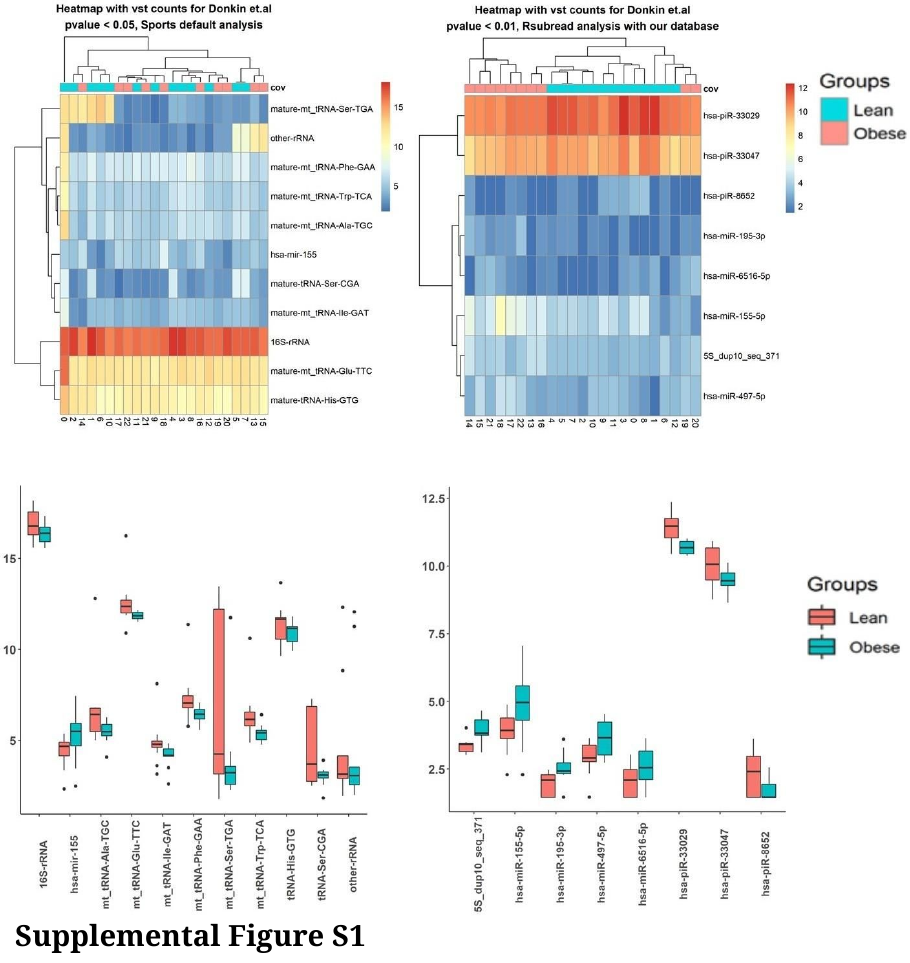

Supplemental Figure S1

## Slide 2
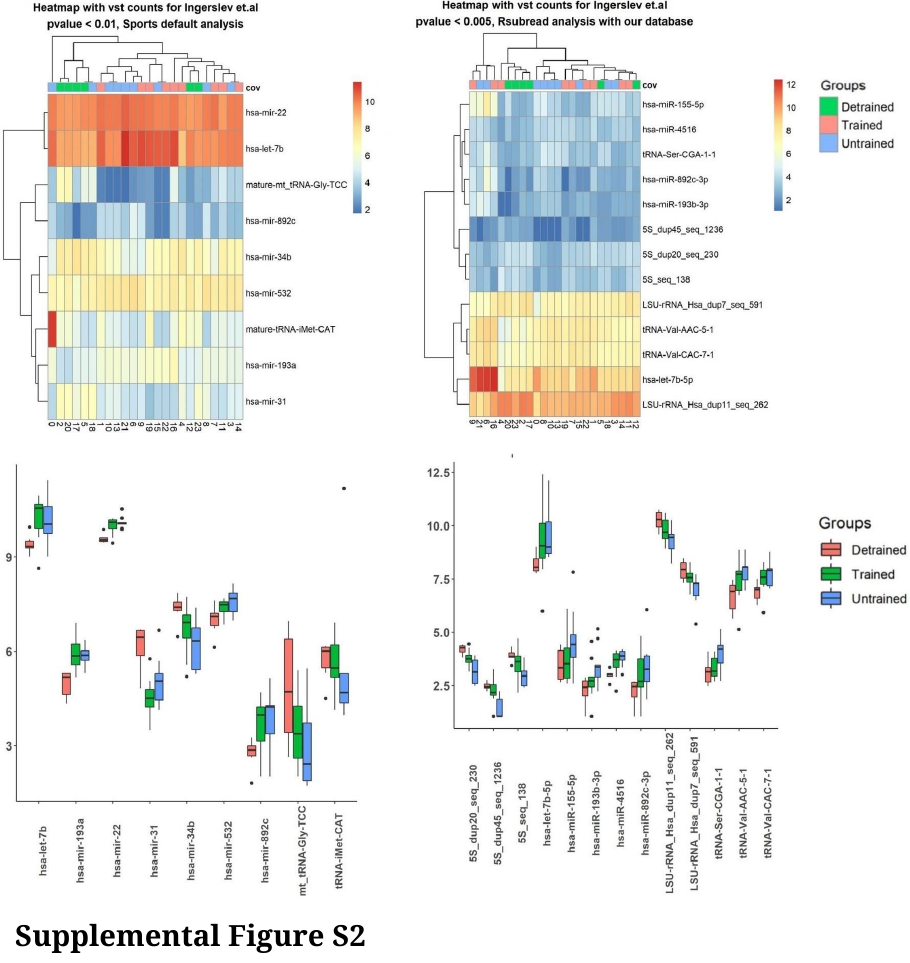

Supplemental Figure S2

## Slide 3
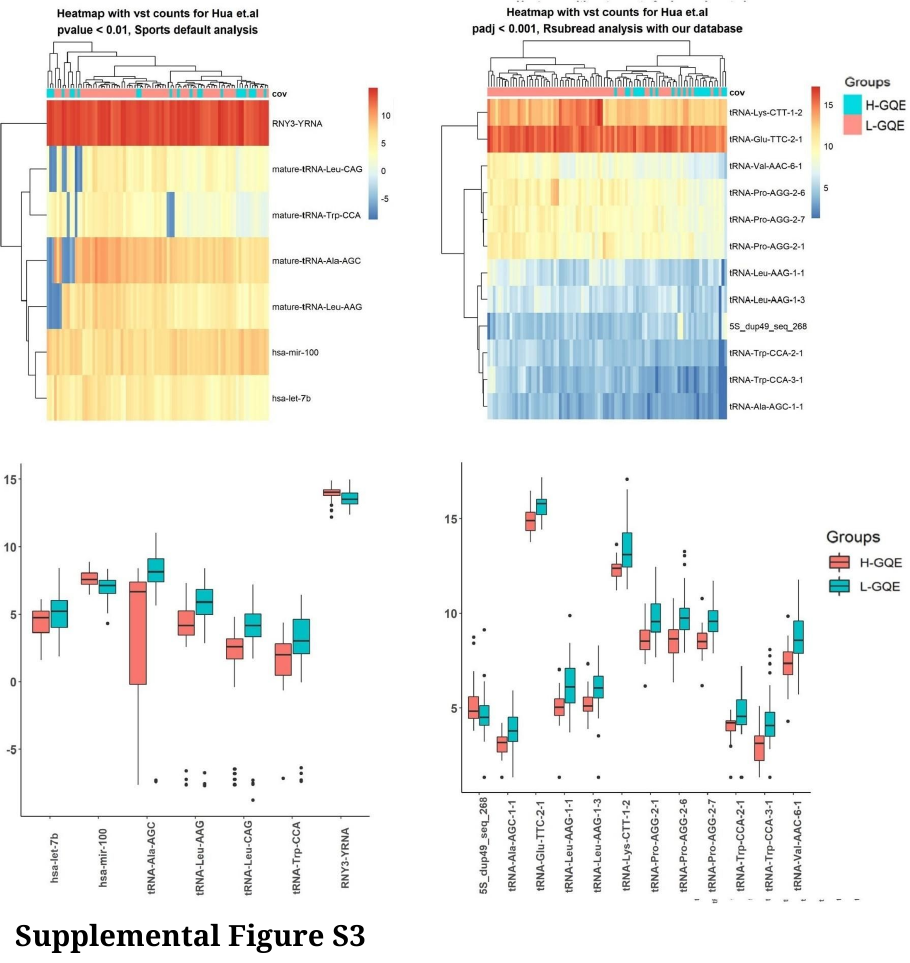

Supplemental Figure S3

## Slide 4
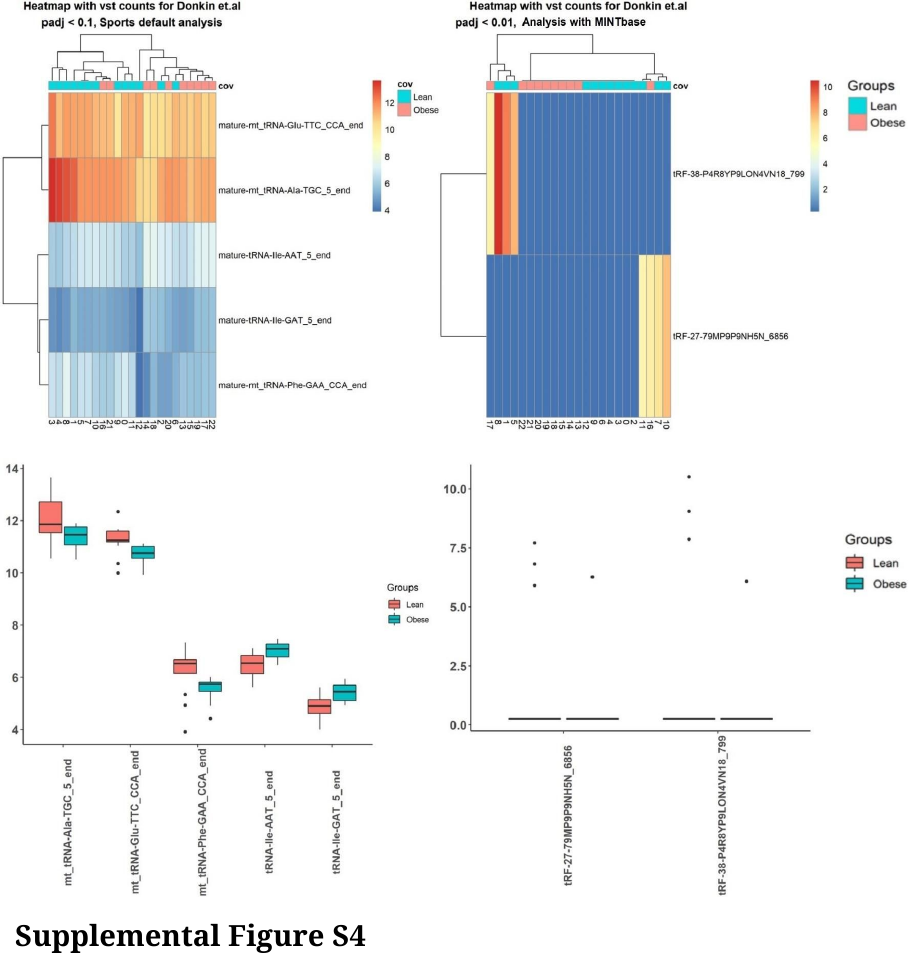

Supplemental Figure S4

## Slide 5
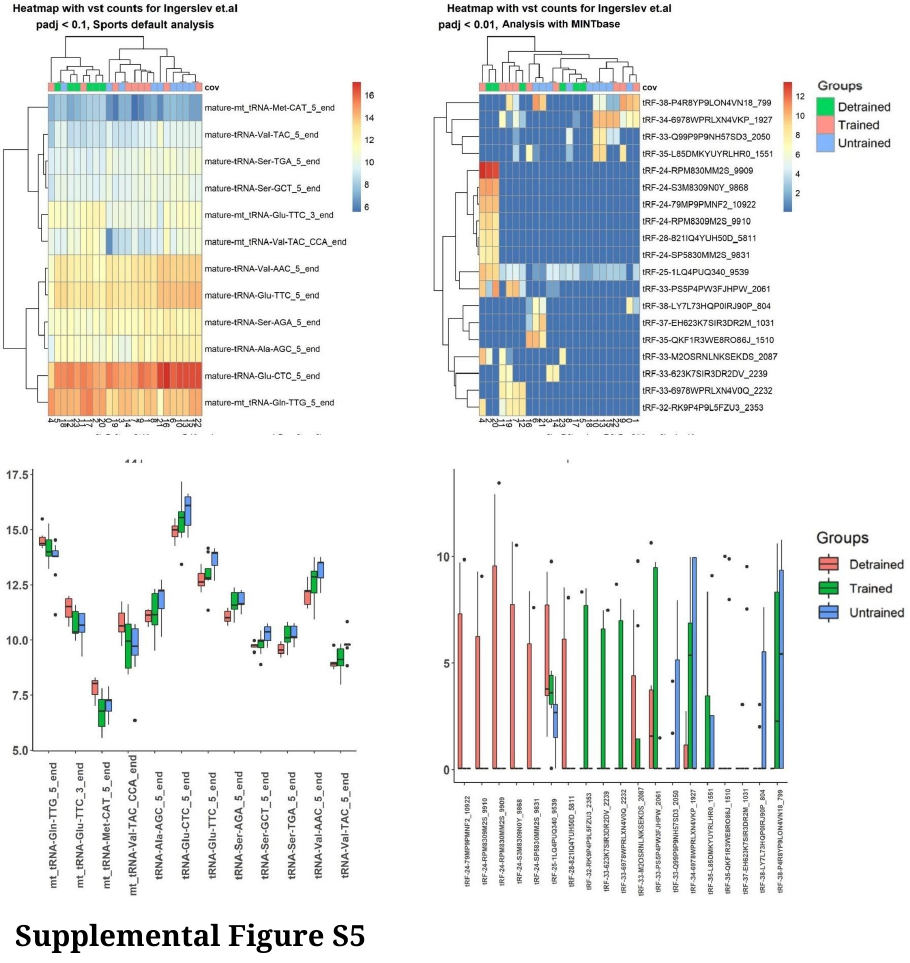

Supplemental Figure S5

## Slide 6
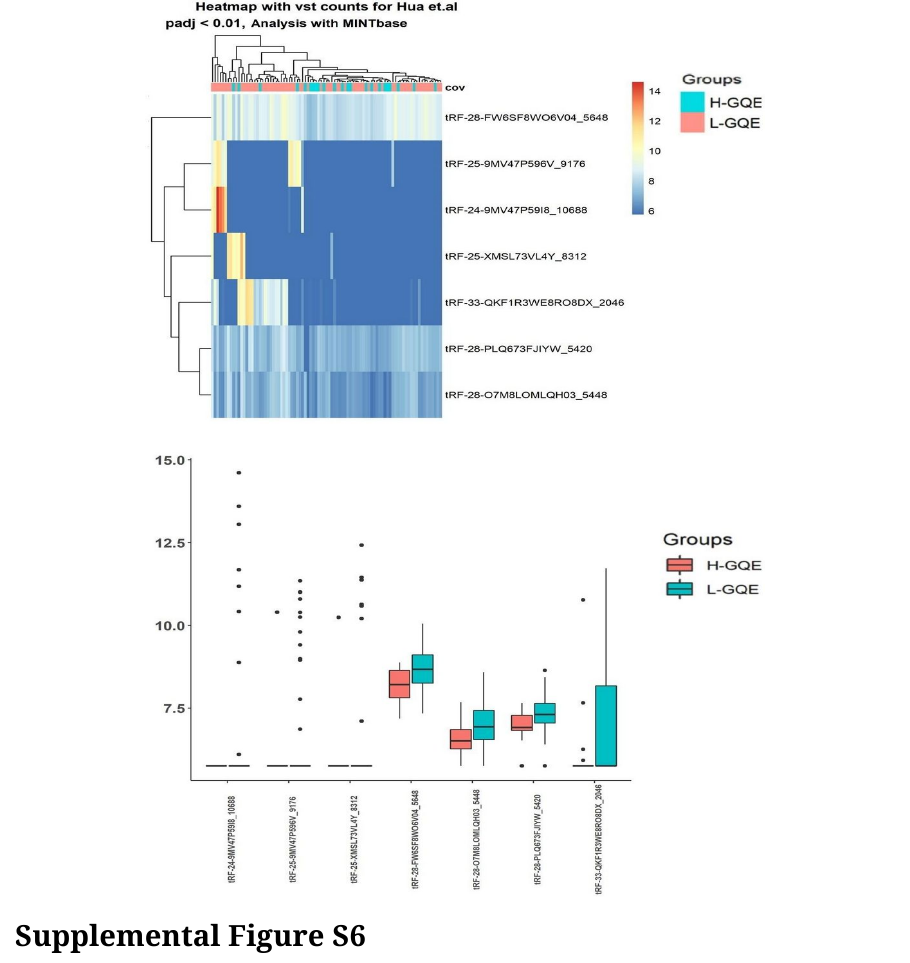

Supplemental Figure S6
